# A novel transformer-based approach for cardiovascular disease detection

**DOI:** 10.3389/fdgth.2025.1548448

**Published:** 2025-04-29

**Authors:** Nimra Noor, Muhammad Bilal, Saadullah Farooq Abbasi, Omid Pournik, Theodoros N. Arvanitis

**Affiliations:** ^1^ Department of Artificial Intelligence, Rare Sense Inc, Covina, CA, United States; ^2^ Department of Electronic, Electrical and Systems Engineering, University of Birmingham, Birmingham, United Kingdom

**Keywords:** electrocardiogram, classification, random forest, cardiovascular diseases, transformers, heart diseases

## Abstract

According to the World Health Organization, cardiovascular diseases (CVDs) account for an estimated 17.9 million deaths annually. CVDs refer to disorders of the heart and blood vessels such as arrhythmia, atrial fibrillation, congestive heart failure, and normal sinus rhythm. Early prediction of these diseases can significantly reduce the number of annual deaths. This study proposes a novel, efficient, and low-cost transformer-based algorithm for CVD classification. Initially, 56 features were extracted from electrocardiography recordings using 1,200 cardiac ailment records, with each of the four diseases represented by 300 records. Then, random forest was used to select the 13 most prominent features. Finally, a novel transformer-based algorithm has been developed to classify four classes of cardiovascular diseases. The proposed study achieved a maximum accuracy, precision, recall, and F1 score of 0.9979, 0.9959, 0.9958, and 0.9959, respectively. The proposed algorithm outperformed all the existing state-of-the-art algorithms for CVD classification.

## 1 Introduction

Cardiovascular diseases (CVDs) are responsible for almost 18 million deaths annually (1). These CVDs mainly consists of arrhythmia (ARR), atrial fibrillation (AFF), congestive heart failure (CHF), and normal sinus rhythm (NSR). There are multiple factors that are responsible for cardiac diseases, i.e., lifestyle, diet, smoking, poor sleep, etc. Changing these conditions can significantly improve heart health. In addition, early prediction and diagnosis of these heart conditions can significantly reduce the number of deaths. Heart disease classification using electrocardiography (ECG) plays an important role in automatic detection of aforementioned diseases.

After the recent advancements in high-powered processors and graphic processing units (GPUs), deep learning (DL) has been increasingly used in the automatic detection of diseases such as epilepsy (2, 3), sleep disorders (4, 5), and heart diseases (6, 7). Incorporating cardiophysiological prior knowledge into the deep neural network architecture improves the performance of automatic detection of CVDs (8, 9). Common considerations include the analysis of PQRST wave patterns, cardiophysiologically meaningful feature extraction, and the temporal dynamics of ECG signals. According to cardiological studies, different segments of the ECG waveform provide critical insights into various heart conditions. Several deep learning methods (10, 11), leveraging this prior knowledge, have achieved superior heart disease detection performance. ECG electrodes are placed on the chest, forming a structured representation of cardiac activity. Consequently, many studies treat ECG signals using techniques such as convolutional neural networks (CNNs) (12) that respect the sequential nature of the data or even graph-based methods for more complex interdependencies (13). Despite these advancements, there remains additional prior knowledge to be explored. As one of the critical physiological processes, heart function involves intricate processes such as electrical conduction, myocardial contraction, and autonomic regulation. Different heart regions exhibit unique activation patterns under various physiological states, for example, the coordinated activity of the atria and ventricles during a cardiac cycle (14) and the interaction between the sinoatrial node and atrioventricular node in maintaining heart rhythm (15).

A predefined or single learnable matrix is unable to capture the intricate connections between different heart parameters that underlie complex cardiovascular conditions. Additionally, it is well-known that heart signal states associated with heart health can fluctuate continuously over short periods but may not remain consistent over extended periods. There have been very limited studies incorporating this temporal context into understanding the effect of this in CVDs. To address this issue, this study developed a novel transformer-based algorithm for CVD classification. Initially, 56 features were extracted from 1,200 ECG samples. To reduce the computational cost and only add the relevant information, the 13 most prominent features were selected using the random forest algorithm. Finally, these 13 features were tokenized and inserted into the proposed transformer model for training. The proposed algorithm outperformed all the existing algorithms for CVD classification with approximately 100% classification accuracy for the four classes.

The main contributions of the proposed study are as follows:

• To reduce the computational cost and increase efficiency, 13 most prominent features were selected from the dataset using the random forest (RF) algorithm.
• A novel transformer-based algorithm was developed for the classification of four classes of CVD.
• Extensive experiments and a comparison has been presented with existing state-of-the-art studies for validation.

## 2 Literature review

This section will briefly explain the literature review and is mainly divided into two parts: CVD classification and transformers.

### 2.1 Transformer neural network

A transformer is a type of deep learning model proposed by Vaswani et al. (16). A transformer offers a significant improvement over CNNs and other existing architectures due to its ability to model long-range dependencies and capture global context through self-attention mechanisms. The self-attention mechanism enables the model to weigh the importance of different input elements dynamically, allowing it to capture more complex and global relationships in the data. Initially, transformer models were used to translate speech and text nearly in real-time. This innovation led to the evolution of large language models such as GPT2 (17) and GPT3 (18). There were two main innovations that the transformer model brought to the market: positional encoding and self-attention. In 2018, bidirectional encoder representations from transformers (BERT) (19) was developed. This revolutionized the large language models, and in 2019, BERT was nearly used for all Google English language searches. In recent years, researchers have proposed different transformer-based algorithms for earthquake detection (20), stock prediction (21), and voltage stability assessment (22). In 2023, these models were explored in biomedical signal classification, including in electroencephalography (EEG) (23) and ECG (24). The property of position encoding and deep self-attention can significantly improve the performance of real-time bio-signal classification, prediction, and diagnosis.

In 2023, Hu et al. proposed a hybrid transformer model (HTM) for epilepsy prediction. The model processes EEG data at multiple levels and uses channel attention to enhance accuracy (25). Their study achieved an optimal sensitivity of 91.7% with a false positive rate of 0.00/h. Automatic sleep apnea (SA) detection using DL and single-lead ECG has been extensively studied. Hu et al. (26) proposed an HTM by exploring the impact of different deep learning model structures and label mapping lengths (LMLs) on personalized transfer learning (TL). The study compared a pure CNN-based model (PCM) with the HTM and evaluated different TL strategies. The results showed that the proposed model achieved an average accuracy of 85.37% and an AUC of 0.9147. The study suggested that increasing LML positively impacts model performance and that using only positive samples is beneficial within the same database, while negative samples are more effective in cross-database TL. However, the study focused only on single-lead ECG data, which may limit its applicability to multimodal approaches. To further improve the personalization of single-lead ECG-based obstructive sleep apnea (OSA) detection, (27) introduced a semi-supervised algorithm for automated fine-tuning. The approach used a CNN-based autoencoder (AE) with an anomaly detection mechanism to assign pseudo-labels to unknown samples, thereby reducing reliance on clinical annotations. The proposed study demonstrated that pseudo-labeling and semi-supervised fine-tuning enhance OSA detection performance while reducing the dependency on annotated clinical data. However, despite these improvements, the approach remains constrained by the limitations of single-lead ECG data and the effectiveness of pseudo-label assignment in highly heterogeneous datasets. Building on the need for enhanced OSA detection, Hu et al. (28) proposed a modality fusion representation enhancement (MFRE) framework to improve diagnostic performance by integrating multiple modalities. Unlike previous single-modal models, this framework used a parallel information bottleneck modality fusion network (IPCT-Net) to extract local–global multi-view representations and eliminate redundant information in fused data. By incorporating multimodal data fusion, this approach addressed the limitations of previous single-modality methods, providing a more robust and clinically relevant AI-assisted OSA screening system.

### 2.2 Cardiovascular disease classification

Considering that there is no definite diagnosis of heart failure, medical diagnostic methods such as assessing the history of the patient, ECG, and echocardiography are crucial for heart disease detection. Of the abovementioned methods, ECG is considered the only non-invasive and cheapest way to assess the health of the heart. Researchers have proposed numerous classification algorithms for detecting cardiac ailments using ECG signals. These include neural networks (NNs) (29), support vector machines (SVMs) (30), decision trees (DTs) (31), and K-nearest neighbors (KNNs) (32). Among these, neural networks, SVMs, and KNNs are particularly prevalent.

Khalaf et al. (30) utilized principal component analysis (PCA) combined with an SVM to classify different types of arrhythmias based on raw spectral correlation data, achieving an accuracy of 98.60%. Thomas et al. (31) applied dual tree complex wavelet transform (DTCWT) for feature extraction and used a multi-layer back propagation neural network to classify cardiac arrhythmias, resulting in a sensitivity of 94.64%, which outperformed the discrete wavelet transform by 3.41%. Escalona-Morán et al. (33) categorized Massachusetts Institute of Technology-Beth Israel Hospital (MIT-BIH) cardiac arrhythmia data into five beat types, achieving a mean accuracy of 98.43%. Christov et al. (34) classified atrial fibrillation signals from a challenge database, obtaining an F1 score of 85% for atrial fibrillation beats.

Acharya et al. (12) proposed a CNN for arrhythmia diagnosis, which automatically classified five different heartbeat types. The algorithm employed data augmentation to balance the dataset, achieving an accuracy of 94% on the balanced data and 89.07% on the imbalanced data. To enhance accuracy further, Long Short-Term Memory (LSTM) networks, a popular and effective model for sequence learning, have been utilized. Darmawahyuni et al. (35) used LSTM and gated recurrent unit (GRU) classifiers to distinguish myocardial infarction (MI) from normal signals in the PhysioNet PTB Diagnostic ECG Database, achieving an accuracy of 97.56% and a Matthews correlation coefficient (MCC) of 95.32% with the LSTM architecture, which outperformed GRU. Oh et al. (36) proposed a hybrid model combining an CNN and LSTM to diagnose five classes from an MIT-BIH dataset. Their model included convolutional, pooling, LSTM, and fully connected layers. The LSTM layers handle the extraction of temporal information from the feature maps created by the convolutional layers. This model achieved an accuracy of 98.10%.

## 3 Materials and methods

**Figure 1:**
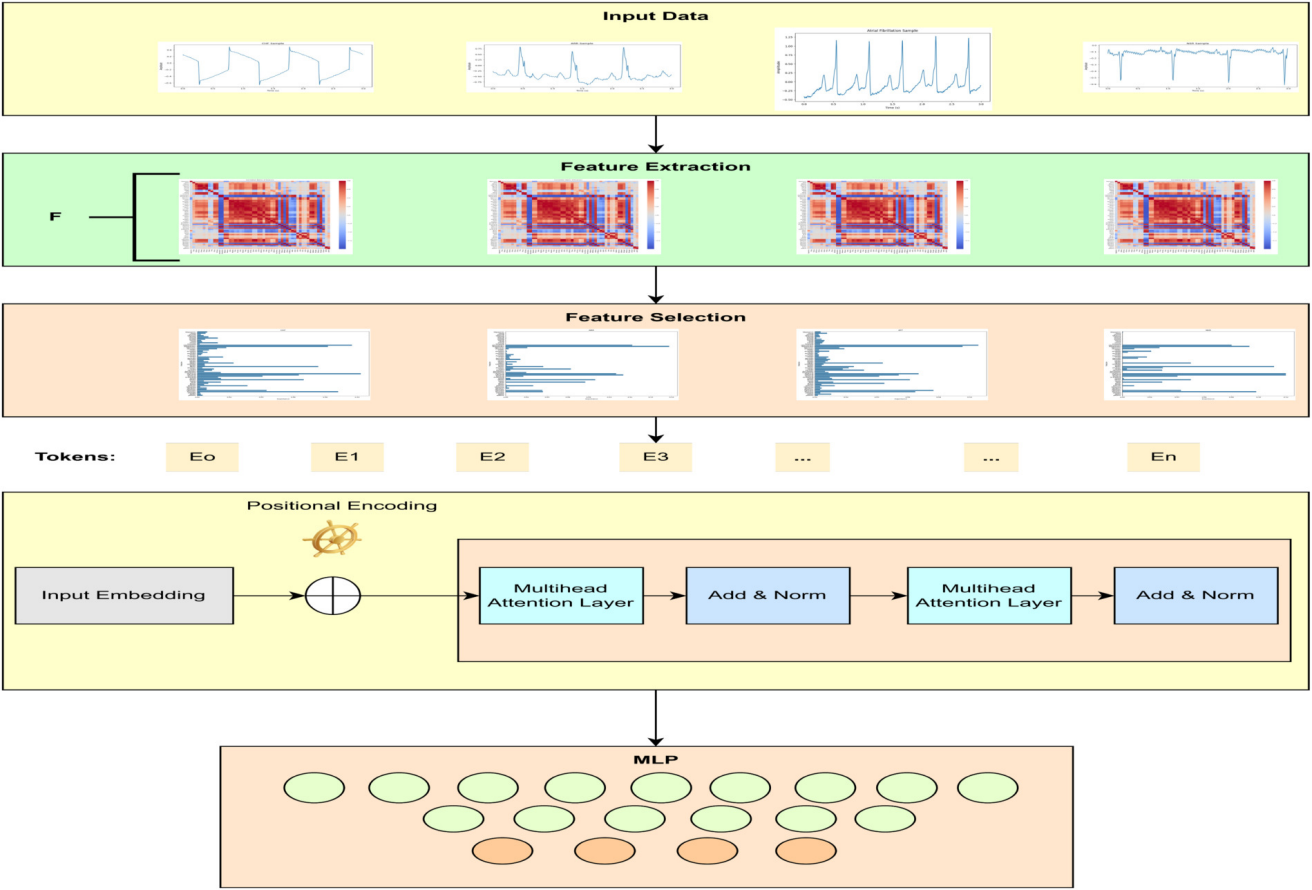
Block diagram of the proposed CVD classifier.

This section briefly explains the dataset, preprocessing, feature extraction and selection, and the proposed transformer model for CVD classification. The block diagram of the proposed transformer-based classifier is given in Figure 1.

### 3.1 Dataset and preprocessing

**Figure 2:**
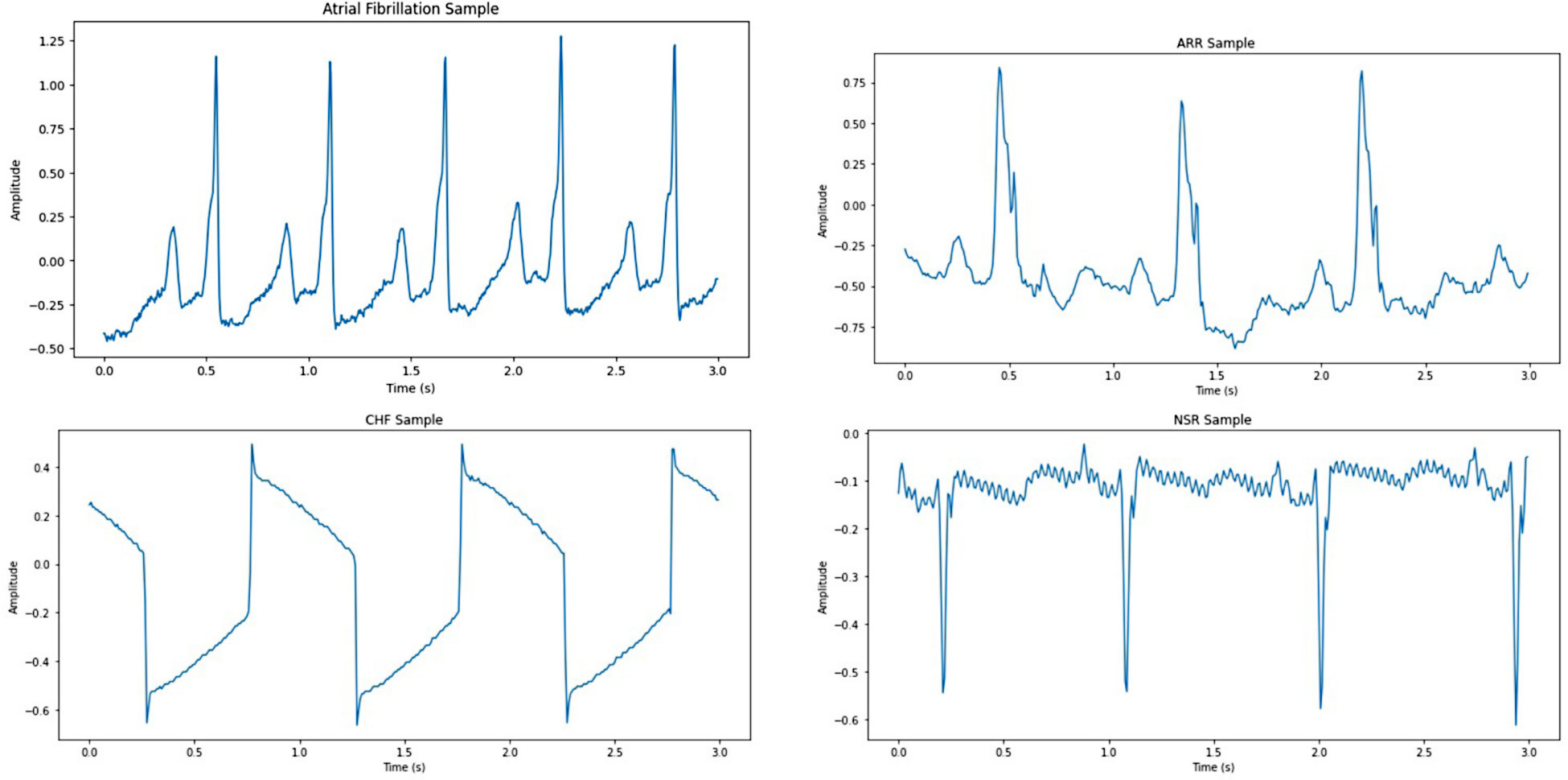
Raw 3 s ECG samples for CVDs.

This study used 1,200 ECG recordings from the MIT-BIH PhysioNet database (37) containing 300 samples for each heart disease, i.e., ARR, AFF, CHF, and NSR. All the recordings were extracted using a sampling rate of 250 samples per second. Raw 3 s ECG recordings after applying the Butterworth filter can be seen in Figure 2. During recording and transmission, these ECG recordings were contaminated with noise and artifacts. To remove noise and artifacts, a Butterworth band-pass filter was applied, ensuring the retention of critical ECG components. The Butterworth filter was chosen due to its maximally flat frequency response in the passband, minimizing distortion. A fourth-order Butterworth band-pass filter with cutoff frequencies of 0.5 and 150 Hz was used to eliminate baseline noise and high-frequency noise.

### 3.2 Feature extraction and selection

The proposed study used maximal overlap discrete wavelet packet transform (MODWPT) to extract characteristics waves, heart rate variability (HRV), and 54 other features. These features are provided in Table 1. Mathematically, wavelet decomposition using MODWPT is given as in Walden and Cristan (38) (Equations 1, 2):

(1) $${\tilde{X}}_{\;j,n,t}^{P}=\sum_{l=0}^{L-1}{\tilde{g}}_{n,l}{\tilde{X}}_{\;j-1,[n/2{]}_{2},(t-2^{\;j-1}l)\;\mathrm{mod}\;\;N}^{P}$$

(2) $$\begin{array}{rl} {\tilde{g}}_{n,l} & =\left\{ \begin{array}{cc} {\tilde{a}}_{l},\; & \text{if }n\;\mathrm{mod}\;\;4=0\text{ or }3;\;\\ {\tilde{b}}_{l},\; & \text{if }n\;\mathrm{mod}\;\;4=1\text{ or }2.\; \end{array}\right. \end{array}$$

where ${\tilde{X}}_{\;j,n,t}^{P}$ are the MODWPT coefficients at time t, which are typically associated with frequencies within the interval $\left(\frac{n}{2^{\;j+1}},\frac{n+1}{2^{\;j+1}}\right]$. The operator [⋅] denotes the integer part (or floor) operator. Four-level symlet transform has been used to detect the characteristic curves. Based on the four-level structure, the signal yields 16 coefficients, with the initial four utilized for signal reconstruction via inverse MODWPT. The peak value of this reconstructed signal corresponds to the R wave. The subsequent characteristic waves are extracted using a suitable moving window technique.

• 1 feature as Heart beat per minute. In total, we have 54 features;
• 11 morphological features;
• 29 fiducial features;
• 4 statistical features;
• 9 HRV features.

**Table 1:** Features from each ECG recording.

Feature type	Features
Morphological features	P duration, QRS duration, T duration, one cycle duration of ECG signal, QRS area, QRS perimeter, and Angles: ∠PQR, ∠QRS, ∠RST, ∠PonPQ, and ∠STToff
Fiducial features	Distance features (permutation of distances between each characteristic wave), slope features (PQ, QR, RS, and ST slopes), and interval features (PQ, PT, QR, QT, RS, and ST segments)
Statistical features	RR mean, PP mean, ratio of QR to QS interval, and ratio of RS to QS interval
Heart rate variability (HRV) features	IBIM, SDRR, IBISD, NN50, pNN50, SDSD, RMSSD, RRTot, and NNTot

Utilizing all 54 features for training and testing will significantly increase the computational cost. There are a number of machine learning methods that can be used to compute the feature importance score. However, we have used RF, a method commonly used for multi-class problems and dealing with dense problems (39). RF is an effective technique that requires minimal parameter tuning. RF consists of multiple binary DTs built on randomly chosen subsets. One crucial characteristic of RF is the use of Out-of-Bag (OOB) error estimation. OOB samples are not used in training the current tree, which allows for internal estimation of generalization error, thereby improving classification performance. This feature is also essential for quantifying feature importance.

RF was chosen for feature selection due to its robustness to noise and outliers, as it can handle noisy or non-linearly separable data efficiently, unlike linear techniques such as PCA (40). RF assigns importance scores to features, ranking them based on their contribution to classification accuracy. Additionally, RF requires minimal parameter tuning compared to methods such as least absolute shrinkage and selection operator (LASSO), and it can handle high-dimensional data effectively by modeling complex interactions between features through an ensemble of decision trees, outperforming individual selection methods such as mutual information or univariate filtering.

RF initially estimates the OOB error of each feature $\text{err}(N^{j})$. It then replaces the feature value with one of its values in the OOB set and re-estimates the OOB error $\text{err}(N_{\text{oob}}^{j})$. The importance score for a feature is defined as the average absolute difference in OOB errors across all trees. Figure 4 shows the feature importance score for each class (Equation 3).

(3) $$VI(N^{j})=\frac{1}{\text{nb\_trees}}\sum_{t=1}^{\text{nb\_trees}}|\text{err}(N^{j}{)}^{\left(t\right)}-\text{err}(N_{\text{oob}}^{j}{)}^{\left(t\right)}|$$

Here, nb_trees represents the number of trees in the RF ensemble. $\text{err}(N^{j}{)}^{\left(t\right)}$ denotes the OOB error of feature $N^{j}$ in the tth tree, and $\text{err}(N_{\text{oob}}^{j}{)}^{\left(t\right)}$ denotes the corresponding error after swapping the feature value.

### 3.3 Proposed methodology

This study proposed a novel transformer-based algorithm for the classification of four classes of CVD. A transformer with token mixers is proposed to capture information from temporal textual information using selected ECG features underlying CVD. This study proposed a token mixer for CVD classification. Since the selected feature dataset consists of numerical feature values and textual labels, we first performed PCA to visualize the data in 2D space and select features with importance greater than 0.02, thereby reducing dimensionality. We then converted each row of features into a textual string format (e.g., “QRtoQSdur: 0.001, RStoQSdur: 0.001…”). The label encoder was used to convert the cardiac condition categories (such as “ARR”) into numerical values for machine learning. These text strings were tokenized using BERT’s tokenizer, which converts them into numerical token IDs that BERT can process. Finally, the tokenized data were converted into PyTorch tensors, and DataLoaders were created for both training and validation.

#### 3.3.1 Transformer layer formulation

Given the feature set $F_{T}=\{f^{i}\}\in R^{\text{len}\times d_{f}}$, a transformer block for ECG classification can be expressed as follows (Equations 4, 5):

(4) $$Y^{n}={\text{TokenMixer}}_{\text{class/reg}}(\text{Norm}(Y^{n-1}))+Y^{n-1},$$

(5) $$Y^{n+1}=\text{MLP}(\text{Norm}(Y^{n}))+Y^{n},$$

where n=[1,2,…] denotes the number of layers in the transformer blocks, $Y^{0}=F_{T}$, and the MLP consists of two linear layers with rectified linear unit (ReLU) activation. Each linear layer is followed by a dropout layer.

#### 3.3.2 Token mixers for classification tasks

For the classification task, the Multi-Head Self-Attention (MHSA) mechanism is used in the TokenMixer. This mechanism emphasizes parts of the feature set $F_{T}$ that are highly correlated with the cardiovascular state. The tokens in $F_{T}$ are linearly projected into multiple groups of key ($K^{i}$), query ($Q^{i}$), and value ($V^{i}$) vectors using learnable parameters (Equation 6):

(6) $$\{Q^{i},K^{i},V^{i}\}={\text{LP}}^{i}(F_{T})=F_{T}W_{\mathrm{kvq}}^{i},$$

The scaled dot-product is employed as the attention mechanism to capture long-term dependencies (Equation 7):

(7) $$\text{Attention}(Q,K,V)=\text{Softmax}\left(\frac{QK^{T}}{\sqrt{d}}\right)V,$$

where d is a scaling factor. The outputs from different heads are stacked together (Equation 8):

(8) $$\text{MHSA}(F_{T})=\{\text{Attention}({\text{LP}}^{0}(F_{T})),\ldots ,\text{Attention}({\text{LP}}^{n_{\text{head}}-1}(F_{T}))\},$$

Considering the temporal nature of ECG signals, a short-time aggregation (STA) layer follows the MHSA to learn long-term contextual information. The STA layer applies a 2D convolution operation (Equation 9):

(9) $$\text{STA}(G_{\text{att}})=\text{Reshape}(\text{Conv2D}(\text{drop}(G_{\text{att}}),K_{\text{conv}}))W_{\text{sta}},$$

where Conv2D(⋅) represents a 2D convolution operation, $\text{drop}(G_{\text{att}})$ denotes dropout, and $K_{\text{conv}}$ is the convolution kernel. The reshaped output is then projected using $W_{\text{sta}}$ (Equation 10):

(10) $${\text{TokenMixer}}_{\text{class}}(F_{T})=\text{STA}(\text{MHSA}(F_{T})).$$

**Figure 3:**
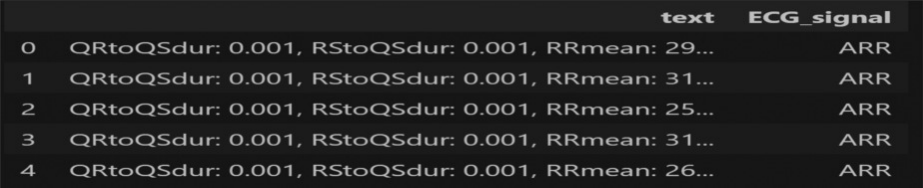
Sample inputs to the proposed transformer model.

A sample input data format for the transformer model is illustrated in Figure 3. Table 2 shows the details of the hyperparameters used in the proposed study. The AdamW optimizer was chosen for its effectiveness in fine-tuning pre-trained models, handling large parameter spaces, and ensuring stable convergence.

**Table 2:** Training hyperparameters.

Optimizer	Learning rate	Epochs	Batch size	Sequence length	Evaluation metric
AdamW	0.00005	5	2	128 tokens	Training loss

### 3.4 Evaluation metrics

The evaluation metrics for CVD classification are mainly divided into four main types: accuracy (Acc), precision (Pre), recall (Rec), and F1-Score. Mathematically, these metrics are given as (Equation 11)

(11) $$\text{Acc}=\frac{1}{N}\sum_{i=1}^{N}\text{I}({\hat{y}}_{i}=y_{i})$$

where

• N is the total number of samples.
• I(⋅) is an indicator function that equals 1 if the condition is true and 0 otherwise.
• ${\hat{y}}_{i}$ is the predicted class.
• $y_{i}$ is the true class.

Precision, recall and F1 score has been calculated for each class. Mathematically, these are given as (Equations 12–14)

(12) $${\text{Pre}}_{c}=\frac{{\text{TP}}_{c}}{{\text{TP}}_{c}+{\text{FP}}_{c}}$$

where

• ${\text{TP}}_{c}$ is the number of true positives for class c.
• ${\text{FP}}_{c}$ is the number of false positives for class c.

(13) $${\text{Rec}}_{c}=\frac{{\text{TP}}_{c}}{{\text{TP}}_{c}+{\text{FN}}_{c}}$$

where

• ${\text{FN}}_{c}$ is the number of false negatives for class c.

(14) $${\text{F1-score}}_{c}=2\cdot \frac{{\text{Pre}}_{c}\cdot {\text{Rec}}_{c}}{{\text{Pre}}_{c}+{\text{Rec}}_{c}}$$

where

• ${\text{Pre}}_{c}$ is the precision for class c.
• ${\text{Rec}}_{c}$ is the recall for class c.

## 4 Results and discussion

**Figure 4:**
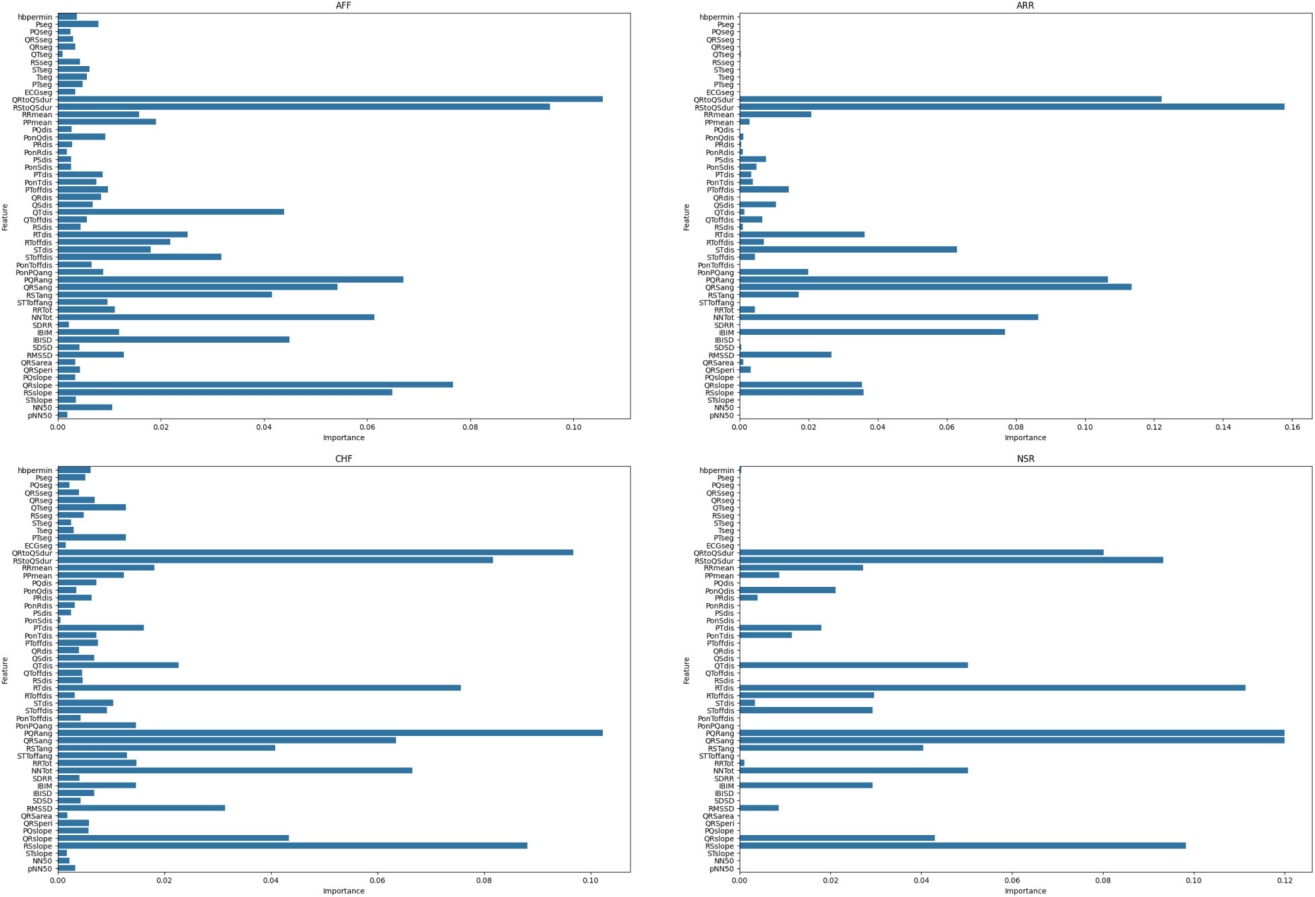
Feature importance score for each heart disease class.

All the experiments were conducted in Python using a 2.50 GHz 12th Gen Intel(R) Core(TM) i9-12900H. In the proposed study, RF was used to calculate the feature importance. First, RF was trained using the training data. The importance of the feature was then calculated and stored. Finally, a reduced number of features were selected on the basis of their importance in each class. The proposed study used a trial-and-error approach, testing various threshold values and selecting 0.02 because it resulted in the highest classification precision while minimizing redundancy. This selection ensured that only the most relevant features were retained for optimal model performance. Figure 4 shows the feature importance scores for each heart disease class.

**Figure 5:**
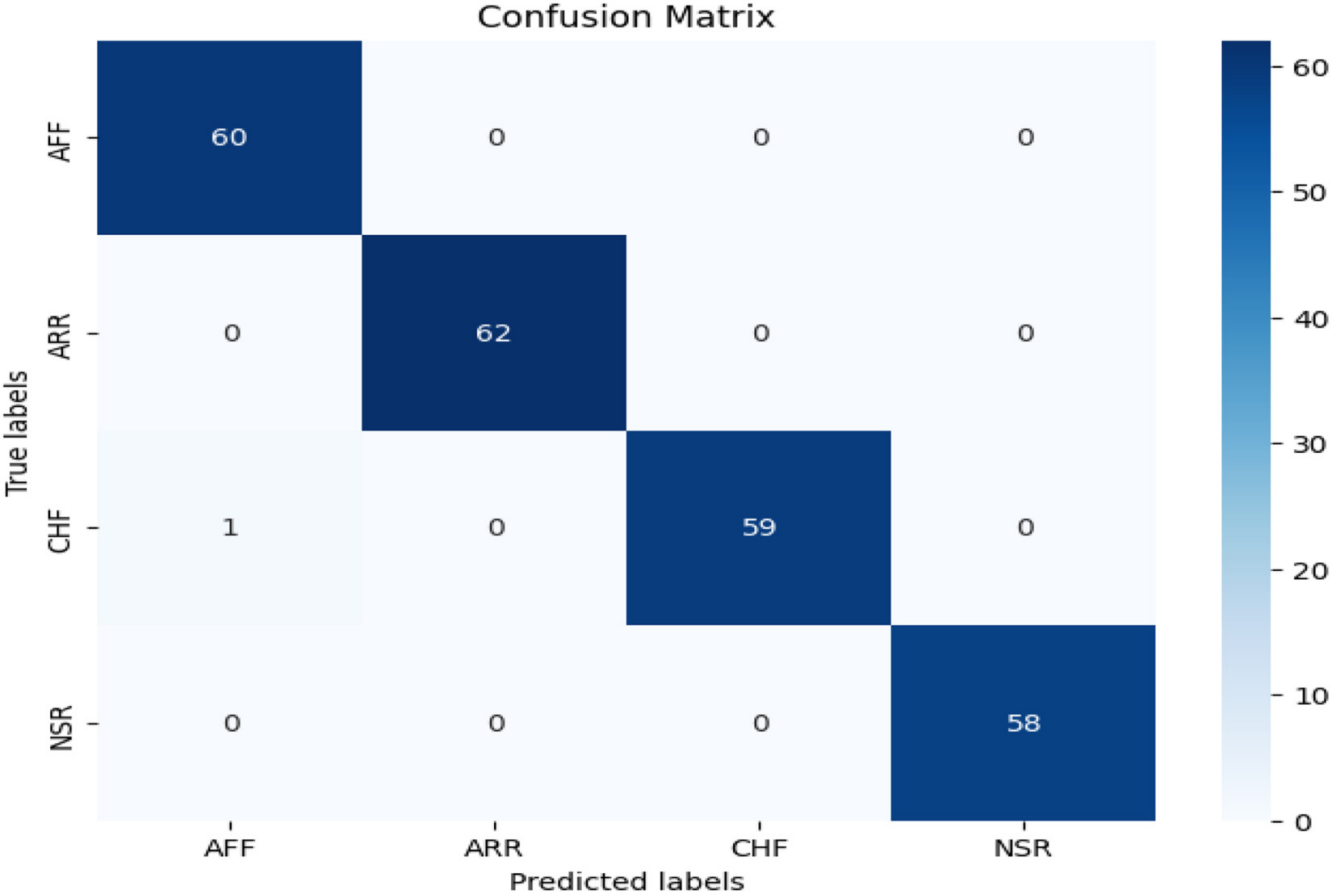
Confusion matrix of the proposed CVD classifier.

The confusion matrix is shown in Figure 5. The evaluation metrics, calculated using the confusion matrix, are also shown in Table 3. We evaluated the proposed study using the evaluation metrics given in Section 3.4. The proposed study achieved an overall notable test accuracy of 99.79%.

**Table 3:** Performance metrics for different cardiovascular conditions.

Performance metrics	Atrial fibrillation	Arrhythmia	Congestive heart failure	Normal sinus rhythm
Acc	0.9958	1.0	0.9958	1.0
Sen	1.0	1.0	0.9833	1.0
Spe	0.9944	1.0	1.0	1.0
Pre	0.9836	1.0	1.0	1.0
F1 score	0.992	1.0	0.9916	1.0

The proposed model avoids overfitting through a 70-10-20 split for training, validation, and testing, to ensure independent evaluation at each stage. The accuracy remained consistent across all three sets, confirming robust generalization without performance degradation. Additionally, to address concerns of overfitting, regularization techniques such as dropout layers and weight decay were applied, ensuring that the model did not rely too heavily on specific patterns.

To demonstrate the necessity of each component in our proposed architecture, we conducted an ablation study by systematically removing or replacing different components. We tested several variations, including a baseline model without feature selection, which used all 56 extracted features without RF-based selection, resulting in an accuracy drop of 2.3%. Additionally, replacing the transformer with a CNN reduced accuracy by 6.28%, highlighting the effectiveness of the transformer model for ECG classification. Furthermore, to validate the claimed improvements, we performed statistical significance tests, comparing our model’s accuracy with existing models such as a multi-layer perceptron (MLP) and a CNN using the paired *t*-test and the Wilcoxon signed-rank test. The paired *t*-test resulted in a *p*-value of 0.0125 for the CNN and 0.0138 for the MLP, indicating that our model’s improvements were statistically significant. The Wilcoxon signed-rank test yielded a *p*-value of 0.0625 for both the CNN and MLP, further confirming the robustness of our method. These results suggest that the improvements observed in our proposed transformer-based approach are not due to random chance but rather to the architectural choices made in this study.

**Figure 6:**
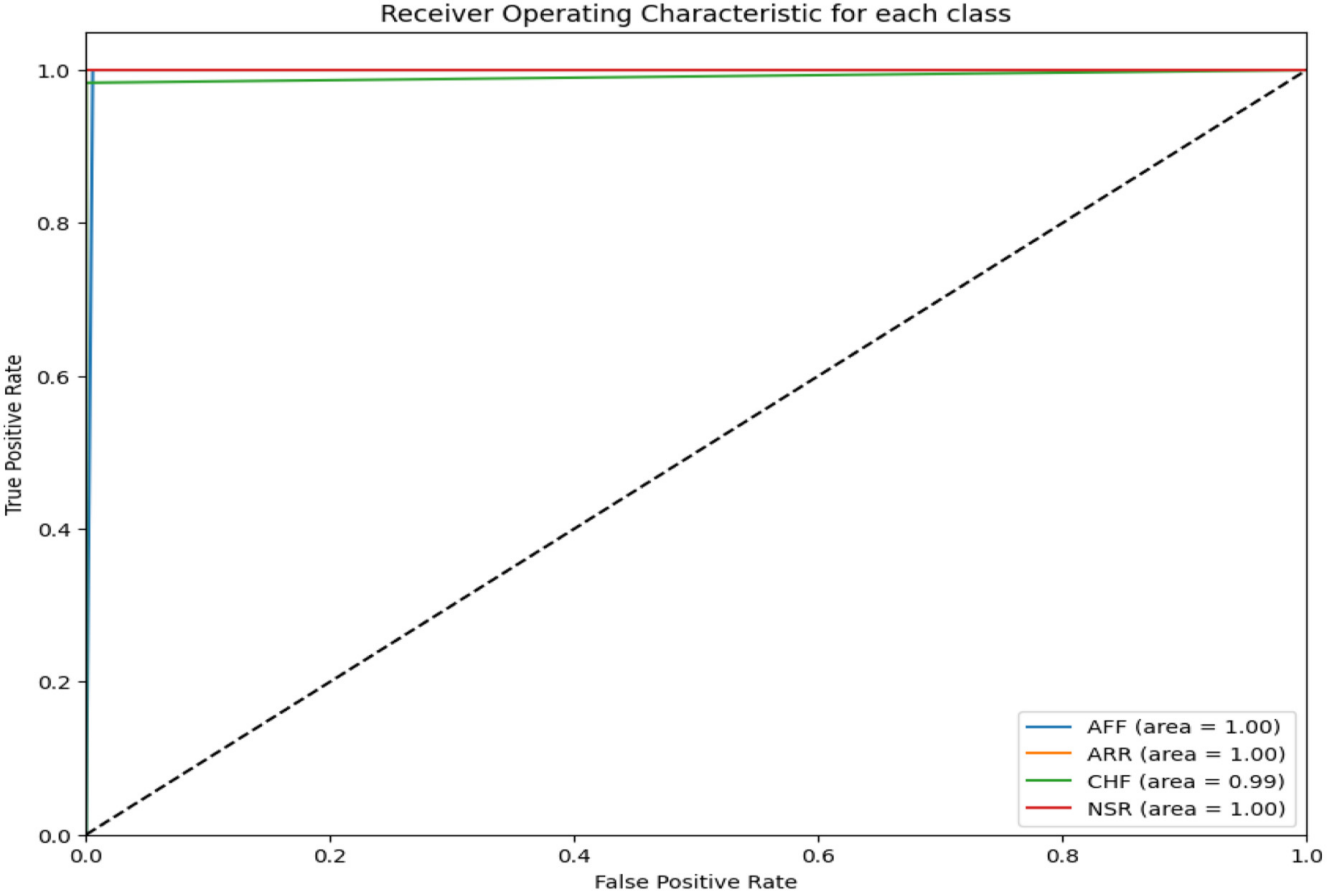
ROC for the proposed CVD classifier.

The area under the receiver operating curve (ROC) provides an aggregate measure of performance across all thresholds. Figure 6 shows the ROC curves for each CVD class on the test set. It can be seen that the area under the ROC is 100%.

In addition to the proposed CVD classification performance, Table 4 provides a comparison with existing algorithms. From the table, it can be seen that the proposed algorithm outperformed all existing algorithms for CVD classification. The results of all evaluation metrics are better compared to the previously proposed algorithms.

**Table 4:** Comparison of different methods and their performance metrics.

References	Method	Performance	Class	Dataset
Çínar and Tuncer (41)	Hybrid model	A = 96.77%	ARR	MIT-BIH (PhysioNet)
	Pre-trained CNN	A = 96.77%	CHF	
	AlexNet—SVM (DWT spectrograms)	A = 100%	NSR	
		A = 96.77%	Overall	
Li et al. (42)	CNN–SVM (Hybrid model)	A = 96.06%	AFF, normal (overall)	West China Hospital, Sichuan University
Qayyum et al. (43)	2D STFT			MIT-BIH (PhysioNet)
	Pre-trained CNN			
	(Hybrid model)		AFF, normal, noisy, other rhythms	
	AlexNet—SVM	A = 93.5%		
	AlexNet—ensemble	A = 91.5%	(overall—multi-class)	
	VGGNet—SVM	A = 97.8%		
	VGGNet—ensemble	A = 96%		
Alekya et al. (44)	2D CWT scalograms		ARR, AFF, CHF, and NSR (overall—multi-class)	MIT-BIH (PhysioNet)
	Pre-trained CNN			
	(Hybrid model)			
	1 VGG16Net	A = 95.3%		
	2 VGG16Net—SVM	A = 95.83%		
	3 VGG16Net—KNN	A = 96.67%		
	4 VGGNet—random forest	A = 96.94%		
Current study	Transformer-based algorithm			MIT-BIH (PhysioNet)
	AFF	A = 99.58%		
	ARR	A = 100%		
	CHF	A = 99.58%		
	NSR	A = 100%		
	Overall	A=99.79%		

STFT, short-time Fourier transform, DWT, discrete wavelet transform; AFF, atrial fibrillation.

To assess the feasibility of deploying the proposed model in clinical settings, we analyzed its computational complexity and real-time performance. The average inference time per ECG sample for the transformer model was 0.022776 s for a batch size of 1, increasing to 0.425322 s for a batch size of 64. This indicates that the model is suitable for real-time applications, especially with smaller batch sizes. The memory footprint of the transformer model, which can be considered a limitation, is approximately 2.656 GB. However, the transformer-based model is optimized for parallel processing on GPUs, allowing efficient handling of large-scale ECG datasets.

The proposed algorithm worked very well for cardiovascular disease detection and classification; however, there are some limitations that need to be addressed. First, the proposed study used four cardiovascular diseases; however, there was no normal class, therefore we could not compare the results of a normal class against each class of cardiovascular disease. The exclusion of a normal class limits the model’s ability to distinguish between healthy individuals and those with cardiovascular diseases, potentially leading to false positives if applied to a dataset containing healthy patients. The proposed dataset used in this study is an dataset available online, and it does not contain a normal class, which is the reason for its exclusion from the proposed model. It is recommended to add a normal class along with the diseases to have a broader and real-world example. Second, the proposed study used hand-crafted features extracted from ECG signals. These hand-crafted features, along with feature selection, require computational time and prior subject knowledge. The proposed model can be extended by integrating a 1D CNN to automatically extract features from raw data. This would allow the proposed architecture to function as a hybrid model, combining automatic feature extraction with advanced classification capabilities, making it adaptable to a broader range of real-world applications.

## 5 Conclusion

The results of the proposed transformer model demonstrate that a transformer-based algorithm can effectively classify four classes of CVD. Initially, 54 morphological, fiducial, statistical, and HRV features were extracted from 3 s ECG data. A random forest algorithm was then used for prominent feature selection. After feature selection, these features were transformed into text and provided as input to the proposed transformer model. The model achieved an impressive accuracy of 99.79%. Due to the absence of a post-processing step, this model is well-suited for real-world applications.

## Data Availability

Publicly available datasets were analyzed in this study. These data can be found here: https://www.kaggle.com/datasets/akki2703/ecg-of-cardiac-ailments-dataset/data.
